# The Microscopic Anatomy of the Human Body in Health and Disease

**Published:** 1847-04-01

**Authors:** 


The Microscopic Anatomy of the Human Body in Health and Disease. Illustrated with numerous Drawings in Colour. By *Arthur Hill Hassall.*

This work, we are glad to find, continues to appear regularly in the monthly Parts, as announced by the author in his prospectus; a point of some consequence to purchasers, when the irregularity of some other works is remembered. The plates are, on the whole, accurate and characteristic; and the text is sufficiently extended to convey a knowledge of the structures represented.

There are, however, some omissions which might, with a little care, be avoided: for example, in the description of the formation and growth of the nails, no notice is taken of what evidently concerns both points, namely, the disposition of the vascular papillæ and loops lying beneath the nail and enclosing both surfaces at the part called the root. Although these organs, the nails, are, as stated in the text, essentially formed of cells, and are therefore extra-vascular, still the disposition of the blood-vessels is a circumstance requiring consideration. The exact extent of the synovial membrane, and the relations of it to the articular cartilage, are not given with the precision of which the subject is now susceptible, and which is required by the great importance of the question in its bearing upon disease. We merely allude to these matters in order that, in the future numbers, every attention should be paid by Mr. Hassall to details of this character. We can again speak in terms of commendation of this work, and doubt not, when completed, that it will be found a very useful compendium of minute anatomy.

